# Short Communication: Evaluation of MALDI-TOF and Sequencing Technique as Typing Tools for *Enterobacteriaceae* Bacteria from Raw Milk of Dairy Cows with Subclinical Mastitis

**DOI:** 10.3390/microorganisms13102267

**Published:** 2025-09-27

**Authors:** Ntelekwane George Khasapane

**Affiliations:** Centre for Applied Food Sustainability and Biotechnology (CAFSaB), Faculty of Health and Environmental Sciences, Life Sciences Department, Central University of Technology, Free State, Bloemfontein 9300, South Africa; nkhasapane@cut.ac.za

**Keywords:** raw milk, MALDI-TOF MS, 16S rDNA, sanger sequencing, *Enterobacteriaceae*, mastitis

## Abstract

Subclinical mastitis is an udder infection and inflammation in dairy animals that causes no visible changes in the milk or udder, making it hard to detect. Animal welfare and health are negatively impacted by dairy cow mastitis, which also severely impairs the dairy industry’s financial standing. This study was carried out in the three local Municipalities of Free State Province, South Africa, sought to determine bacterial contamination of raw milk of cows infected with subclinical mastitis using matrix-assisted laser desorption ionization-time of flight mass spectrometry (MALDI-TOF MS) and 16S rRNA Sanger sequencing. From subclinical mastitic samples, our MALDI-TOF results revealed coliform bacteria such as *Pseudomonas oryzihabitans* with 25/71 (32.21%) as a dominant species, followed by *Pseudomonas aeruginosa*, *Escherichia coli*, *Pantoea agglomerans* and *Enterobacter kobei* at 9/71 (12.67%), 8/71 (11.27%), 6/71 (8.45%) and 6/71 (8.45%), respectively. Finally, *Enterococcus faecalis*, *Enterococcus faecium*, *Micrococcus luteus*, *Routella ornithinolytica* were detected at 3/71 (4.22%) each and 1 sample with mixed species of *Routella platicola* 1/71 (1.40%) and *Routella ornithinolytica* at 1/71 (1.40%). The most frequent microbes causing dairy cow mastitis can be identified using MALDI-TOF MS, a technique that is strong, quick, and accurate. With the inclusion of new species, the database can be enhanced and expanded over time.

## 1. Introduction

Worldwide, the most prevalent and expensive dairy cow illness is mastitis, which is an inflammation due to infection of the mammary gland. It has a negative impact on the milk production industry, resulting in significant financial losses from lower milk production levels in terms of both quantity and quality [1]. In addition to significantly reducing milk production, the subclinically afflicted animals continue to infect other members of the herd [2]. According to certain research, the prevalence of subclinical mastitis in cows varied between 19.20 and 83% [3]. Given its intricacy and the variety of causative microorganisms, it is likewise a complicated disease. Currently, however, total eradication is not possible [4]. Understanding the key risk variables linked to management strategies is therefore crucial for reducing the incidence of subclinical mastitis in dairy cattle. It does not cause any obvious alterations to the udder or milk [3]. Cows with subclinical intramammary infections (IMIs) give less milk and with lower quality, even though the milk looks normal [5]. Milk production can drop by 10% to 20% as a result of subclinical mastitis. Furthermore, it adversely affects the milk’s nutritional value and ingredients, making it of low quality and less suitable for processing [6,7]. Special diagnostic procedures are needed to detect subclinical mastitis because there are no outward signs of abnormalities in the milk [8,9]. Because udder tissue changes occur before they become noticeable, early identification of mastitis—and subclinical mastitis in particular—is crucial [9]. It cannot be identified without a laboratory or field test and mostly remains unnoticed by the farmer, therefore considered a hidden form of mastitis.

Moreover, *Enterobacteriaceae* are one of the few common causal pathogens of mastitis, accounting for the majority of cases. Since raw milk is essentially sterile, contamination can occur in the mammary glands, outer skin, feces, milking machines, during handling, and during storage [10,11]. In the digestive tracts of all vertebrates are facultative anaerobes called coliforms or *Enterobacteriaceae*. *Citrobacter freundii*, *E. coli*, *E. agglomerans*, *E. aerogenes*, and *E. cloacae* are a few possible examples of these species [12]. Intramammary antibiotic therapy is commonly used to treat cases of mastitis once they are identified. However, due to the delayed identification of bacterial pathogens and their antimicrobial sensitivities due to the time required for culture/PCR and antibiogram results prevented the immediate, targeted treatment of subclinical mastitis in the cows [13]. To find these foodborne pathogens in food and safeguard the public’s health, a number of culture-dependent and culture-independent approaches have been developed [14]. The most common methods for identifying bacteria are microbiological ones, like examining the isolates’ morphological and biochemical traits, or, more recently, molecular biology techniques like PCR combined with Sanger sequencing, which targets the 16S rRNA gene, and comparing the isolates’ gene sequences with classified references in popular databases [15]. As a result, the matrix-assisted laser desorption ionization-time of flight mass spectrometry (MALDI-TOF MS) has become a popular alternative to microbiological identification due to its advantages over molecular identification methods and biochemical-based tests in terms of speed, cost, and labour savings [15,16]. Hence, this study employed MALDI-TOF MS and 16S rRNA Sanger sequencing for the identification and characterization of *Enterobacteriaceae* from raw milk of dairy cows with subclinical mastitis.

## 2. Materials and Methods

**Figure 1:**
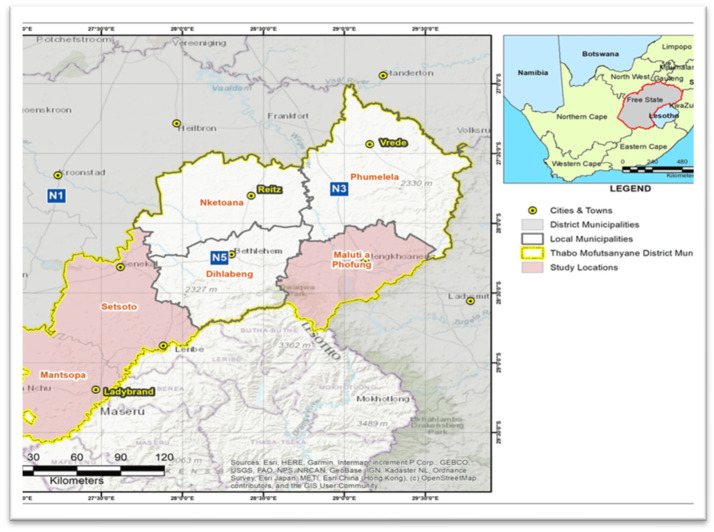
Map showing study area. Pink depicts Mantsopa, Setsoto, and Maluti-a-Phofung local Municipalities in the Free State Province.

The current cross-sectional study was conducted in seven small-scale farms from Maluti-A-Phofung, Mantsopa and Setsoto local Municipalities of the Free State Province in South Africa (Figure 1), which use mechanical milking systems. The sample size in this study was estimated using the formula by Thrusfield [17].

$$n=\frac{1.96^{2}\times P_{exp}(1-P_{exp})}{d^{2}}$$

where n = required sample size, P_exp_ = expected prevalence, and d^2^ = desired level of precision at 95% confidence interval.

To avoid cross-contamination, each cow’s udder was cleaned with distilled water and wiped with a disposable paper towel before sample collection. Before pure milk samples could be taken using 50 mL sterile Falcon tubes, 70% ethanol was administered to each udder to guarantee aseptic sample collection. The original milk streaks were thrown away. Using flow cytometry (Mérieux NutriSciences, Pretoria, South Africa) and the somatic cell count (SCC) assay, 166 composite milk samples from individual cows from seven small-scale farms spread across three Local Municipalities—Maluti-a-Phofung, Mantsopa, and Setsoto—were randomly screened for intramammary infection (Figure 1). According to the manufacturer’s instructions (DeLaval, Free state, South Africa), only 220 individual quarters from 55 out of 166 cows were then subjected to the California Mastitis Test (CMT) on the farm based on the SCC results. Only 160 quarter milk samples were then collected in duplicate using sterile 50 mL Falcon tubes, one batch for a second round of the somatic cell count (SCC) assay (Mérieux NutriSciences, Pretoria, South Africa) and another batch for microbiological analysis. The CMT results were scored and interpreted as 0 (negative [healthy quarter], somatic cell count [SCC] ≤ 100,000 cells/mL milk), 1+ (weak positive, SCC > 100,000 < 500,000 cells/mL milk), 2+ (distinct positive, SCC > 500,000 < 1000,000 cells/mL milk), and 3+; (strong positive, SCC ≥ 1000,000 cells/mL milk) as recommended by Karzis et al. [18]. Preliminary identification of the isolates was done using the RapID ONE kit (Thermofischer, Johannesburg, South Africa) and Gram staining.

### 2.1. Bacteriological Analysis

To do microbiological examination, 100 μL aliquots of the milk samples were inoculated onto Violet Red Bile agar (VRB) using a mug for *Enterobacteriaceae* isolation. After that, the samples were incubated at 37 °C for 24 h. A colour shift to crimson is indicative of lactose-positive colonies, which include *E. coli* and other coliform organisms [19]. UV fluorescence was used to demonstrate *Escherichia coli*. Two to three suspected coliform colonies were then sub-cultured on Nutrient Agar plates (NAP) and incubated at 37 °C for 24 to 48 h in order to establish pure cultures.

### 2.2. Identification of Isolates Using MALDI-TOF Method

Biotyper 3.1 was used to identify isolates or genera of *Enterobacteriaceae* from an outsourced and distributed institution, the University of Pretoria (Bruker, Johannesburg 2191, South Africa). The Autoflex Speed apparatus (Bruker Daltonics, Billerica, MA, USA) was calibrated using the *Escherichia coli* DH5α Bacterial Test Standard (BTS) [19]. In short, sterile 1.0-μL disposable plastic inoculating loops were used to transfer the pure colony to individual 1.5-mL microcentrifuge Safe-Lock Tubes (Sigma-Aldrich, Brondbyvester, Denmark) that contained 300 μL of ultra-HPLC grade water (Merck, Hellerup, Denmark). After briefly vortexing the tubes to produce a uniform suspension, 900 μL of 100% ethanol (Merck) was added, and the tubes were vortexed for a further 15 s. After centrifuging the tubes for three minutes at 12,200× *g* at room temperature (RT), the supernatants were drained out and disposed of, and the tubes were centrifuged once more for three minutes at RT. A micropipette was used to properly aspirate the leftover ethanol/water. After allowing the cell pellets to air dry for three minutes, a suitable volume (15–50 μL) of 70% formic acid was added. By visually sizing the pellet, the ideal formic acid volume was identified. Each sample was thoroughly mixed with an equal volume of 100% acetonitrile after up to three minutes. At room temperature, samples were centrifuged for three minutes at 12,200× *g*. After carefully placing eight 1.0 μL volumes of supernatant on a ground steel target plate and letting it air dry, 1.0 μL of cyano-4-hydroxycinnamic acid (HCCA) matrix (Bruker Daltonics) diluted in 50% acetonitrile with 2.5% trifluoroacetic acid (Sigma-Aldrich) was applied to each spot. Every isolate’s analysis was performed twice. If an isolation could not be resolved after two rounds of MALDI-TOF MS analysis, it was considered unidentified. A cut-off score of ≥2.300 was employed as a threshold for the detection of bacteria in order to ensure the credibility of our analysis.

### 2.3. Extraction of Genomic DNA

Additionally, the manufacturer’s instructions for the Quick-DNATM Fungal/Bacterial Miniprep Kit (Zymo Research, Irvine, CA 92614, USA) were followed in order to extract genomic DNA from the appropriate isolates. The 16S rDNA PCR assay was used to identify the species using primers 27F 5′-AGAGTTTGATCCTGGCTCAG-3′ and 1492R 5′–85 TACCTTGTTACGACTT-3′. For the purpose of this paper, PCR mixture contained 1 μL of each forward and reverse primers, OneTaq 2× master mix (4.25 μL) (New England Biolabs, 87 Massachusetts, United States, DNA template (2 μL), and double distilled water (4.25 μL) were used for the amplification. The following conditions were used: 94 °C for 5 min, 50 °C for 30 s after 94 °C for 30 s, 68 °C for 1 min for 35 cycles, and 68 °C for 10 min before being maintained at 4 °C. The PCR amplicons were visualized on a 1% agarose gel (Csl-ag500, Cleaver Scientific Ltd., Rugby, UK). Thereafter, 1 Kb DNA Ladder (Sigma, Cape Town, South Africa. D7058) was utilized as a molecular marker. The 16S rRNA PCR products were sent for Sanger sequencing at Inqaba Biotechnical Industries (Pty) Ltd., Pretoria, South Africa.

## 3. Results

Finding the right mastitis-causing bacteria is crucial for the dairy industry’s treatment and management of dairy cow mastitis. An improper management approach may be used for a particular instance as a result of the consequences of a wrong diagnosis. That being said, a lot relies on the organism [20]. The results of this study revealed that, from a total of 166 milk samples, only 87 (54%) samples were able to grow 71 *Enterobacteriaceae* bacteria, which were identified by MALDI-TOF MS. *Pseudomonas oryzihabitans* and *Pseudomonas aeruginosa* were the dominant species identified with 25 (35%) and 9 (12.67%), followed by *Escherichia coli* at 8 (11.26%), *Enterobacter kobei* and *Pantoea agglomarans* each with 6 (8.45%). Furthermore, the least identified species were *Kosakonia cowanii* 4 (5.63%), *Enterococcus faecalis 3* (4.22%), *Enterococcus faecium* 3 (4.22%), *Raoutella planticola*, *Raoutella ornithinolytica* 3 (4.22%), *Micrococcus luteus* 3 (4.22%), and mixed isolates of *Mesobacillus thioparans* and *Mesobacillus subterraneous* 1 (1.40%) (Table 1) (Supplementary Files S1 and S2). A representative number of 41 isolates were further confirmed using 16S rDNA Sanger sequencing and all isolates were given accession numbers listed below: *Pseudomonas oryzihabitans* [OR461677, OR461678, OR461679, OR461680, OR461681, OR461682, OR461683, OR461684, OR461685, OR461686, OR461687, OR461688, OR461689, OR461690,OR461691, OR461692, OR461693, OR461694, OR461695, OR461696, OR461697], *Pseudomonas aeruginosa* [OR461698, OR461699, OR461700, OR461701, OR461702, OR461703, OR461704, OR461705, OR461706]. While other coliforms were given the following accession numbers [OR540496, OR540497, OR540498, OR540499, OR540500, OR540501, OR540502, OR540503, OR540504, OR540505, OR540506].

## 4. Discussion

*Enterobacteriaceae* is a family of bacteria that can cause subclinical mastitis, which is an inflammation of the mammary gland in dairy cattle. Subclinical mastitis affects milk output and can result in financial losses even if it does not exhibit the same outward symptoms as clinical mastitis. These infections are frequently caused by *Enterobacteriaceae*, which include *Klebsiella pneumoniae* and *Escherichia coli* [21]. This current study was carried out to evaluate the various techniques for identification of subclinical mastitis-causing Enterobacteriaceae in the Free State Province of South Africa. The isolated *Enterobacteriaceae* species were consistent with those of Savage et al. [22], who identified *E. coli*, *Enterobacter*, *Klebsiella* spp., and *Citrobacter* as *Enterobacteriaceae* contamination of milk. It should be emphasized that the presence of *Enterobacteriaceae* was linked to a decline in raw milk quality and consequently to inadequate microbiological safety. Our findings were similarly consistent with those of Ntuli et al. [23], who identified *Klebsiella* spp., *Raoultella ornithinolytica*, and *E. coli* as the most prevalent representatives of the *Enterobacteriaceae*.

Given that *Pseudomonas oryzihabitans* is rarely identified as a major mastitis pathogen, its predominance is especially remarkable. *Escherichia coli*, *Pseudomonas aeruginosa*, *Klebsiella* species, and *Staphylococcus aureus* are the main causes of mastitis globally, according to the majority of prevalence studies [24,25]. The emergence of *P. oryzihabitans* as a dominant isolate in subclinical mastitis raises the possibility of environmental reservoirs or farm-level practices that favor colonization in this area, despite the fact that it has been reported to occasionally cause infections in humans and animals, including wound and skin infections [26,27]. In keeping with its well-established function as an environmental mastitis pathogen, *Pseudomonas aeruginosa* was also found [28]. Biofilm formation in water lines, milking equipment, and tainted teat dips has been connected to its persistence in dairy herds [29]. The rate of infection seen here is consistent with findings from other areas, suggesting that *P. aeruginosa* continues to be a significant pathogen in the event of poor hygiene [28].

Additionally, coliforms such *E. coli* (11.3%), *Enterobacter kobei* (8.5%), and *Pantoea agglomerans* (8.5%). These results are consistent with observations from around the world that coliform bacteria are common causes of both clinical and subclinical mastitis, and that they are frequently connected to unsanitary conditions and tainted bedding [29]. Its reduced prevalence in our dataset, however, indicates regional variation or sample selection bias towards subclinical infections, in contrast to many studies where *E. coli* is the major coliform [30]. The environmental origin of the infections found in this investigation is supported by the identification of *Pantoea agglomerans*, formerly known as *Enterobacter agglomerans*, as an opportunistic mastitis pathogen linked to contaminated plant debris and bedding [31,32,33]. The increasing identification of enterococci in bovine mastitis is reflected in the presence of *Enterococcus faecalis* and *Enterococcus faecium* (4.2% each). Because they might contain antibiotic resistance determinants that are transferable throughout the dairy chain, these organisms, which have been reported worldwide, are concerning [32,34]. The significance of the upcoming antimicrobial susceptibility profiling is highlighted by its discovery in raw milk. The opportunistic aspect of mastitis microbiology, where non-classical organisms might contribute to intramammary infections under particular conditions, is also shown in the sporadic isolation of *Micrococcus luteus* and *Raoultella* spp. [33,34].

Using 16S rDNA sequencing as the standard method, Nonnemann et al. [35] investigated the identification of 500 udder pathogenic isolates by MALDI-TOF and reported that 100% and 93.5% of the bacteria studied were accurately identified to the genus and species levels, respectively. Another study assessing the test agreement between biochemical methods, the MALDI-TOF MS system, and 16S rDNA sequencing for the identification of 181 milk isolates found that the biochemical method had 95% agreement with each of the other two methods, while MALDI-TOF had 98% agreement with 16S rDNA sequencing [36]. We found that MALDI-TOF was effective in detecting Gram-negative isolates at the genus level in our investigation. The restricted reference library available for this bacterial group from dairy cow mastitis sources may be the reason for the lower percentage of correctly identified species among Gram-negative isolates. Furthermore, underrepresentation may result in incorrect or unreliable identification by MALDI-TOF, even if a species is present in the MALDI library.

The MALDI-TOF microbial proteins, which are frequently very conserved within a species, are measured by mass spectrometry. Because of this, it offers a more dependable way to differentiate between species than the conventional methods of microbiological and biochemical identification of bacteria [37]. Additionally, the more than 7000 reference spectra in the MALDI Biotyper database appear to vary based on the isolates’ source, such as environmental, animal, or dietary [38]. By comparing the ten closest reference spectra, the MALDI Biotyper algorithm determines the log score, or distance, between the sample and reference spectra; a greater distance leads to an incorrect identification. Consequently, these naturally occurring milk isolates that were only partially identified (by genus) would have deviated significantly from the reference spectra (from a different source) in the database, leading to an inaccurate species identification (log score < 2.0). This finding is consistent with research by Savage et al. [22] that proposed improving the reference library for certain genera and ascribed partial identification (genus only) to database limitations.

Whereas 16S RNA Sanger sequencing was unable to clearly identify certain species, MALDI-TOF MS was able to detect all bacteria down to the species level. This is because of the combined results of the few studies that have been done so far, which demonstrate that 16S rRNA gene sequencing typically identifies the genus but not the species, with 1–14% of isolates remaining unidentified following testing [39,40]. Recently identified taxa, a lack of sequences in nucleotide databases, species with similar or identical 16S rRNA sequences, or problems with nomenclature resulting from multiple genomovars belonging to a single species or complex can all make it challenging to identify a genus and species [41,42]. Hence, a customized database containing dairy cow mastitis-sourced bacteria will be more efficient in identifying the mastitis pathogens at the genus and species level. As part of the limitations, the current study did not take into account the breeds of dairy cows, animal husbandry practices, milking systems, milk yield, and age of the animals.

## 5. Conclusions

In dairy cows from the Free State Province, this study identifies a wide range of bacterial species linked to subclinical mastitis, with *Pseudomonas oryzihabitans* being the most common isolate. The identification of opportunistic pathogens such as enterococci and coliforms draws attention to the hidden cost of subclinical infections as well as the possible effects on public health, food safety, and milk quality. Our findings establish a basis for more precise and prompt surveillance of mastitis infections by highlighting the importance of sophisticated diagnostic techniques like MALDI-TOF MS and 16S rRNA sequencing. Reducing financial losses and ensuring safer dairy production necessitates a concerted effort that incorporates industry-level reforms, better on-farm practices, and veterinarian health management.

## Figures and Tables

**Table 1:** Identified bacteria from subclinical dairy cow mastitis by MALDI-TOF MS.

Bacteria	Log Score	Bacteria	Log Score	Bacteria	Log Score	Bacteria	Log Store	Bacteria	Log Score	Bacteria	Log Score	Bacteria	Log Score	Bacteria	Log Score
*E. faecalis*	≥2	*E. coli*	≥2	*P. oryzihabitans*	≥2	*P. oryzihabitans*	≥2	*P. oryzihabitans*	≥2	*P. aeruginosa*	≥2	*P. agglomarans*	≥2	*M. thioparans*	≤2
*E. faecalis*	≥2	*E. coli*	≥2	*P. oryzihabitans*	≥2	*P. oryzihabitans*	≥2	*P. oryzihabitans*	≥2	*E. faecium*	≥2	*P. agglomarans*	≥2	*M.subterraneuos*	≤2
*E. faecalis*	≥2	*E. coli*	≥2	*P. oryzihabitans*	≥2	*P. oryzihabitans*	≥2	*P. aeruginosa*	≥2	*E. faecium*	≥2	*P. agglomarans*	≥2		
*E. kobei*	≥2	*E. coli*	≥2	*P. oryzihabitans*	≥2	*P. oryzihabitans*	≥2	*P. aeruginosa*	≥2	*E. faecium*	≥2	*R. planticola*	≥2		
*E. kobei*	≥2	*E. coli*	≥2	*P. oryzihabitans*	≥2	*P. oryzihabitans*	≥2	*P. aeruginosa*	≥2	*K. cowanii*	≥2	*R. ornithinolytica*	≥2		
*E. kobei*	≥2	*E. coli*	≥2	*P. oryzihabitans*	≥2	*P. oryzihabitans*	≥2	*P. aeruginosa*	≥2	*K. cowanii*	≥2	*R. ornithinolytica*	≥2		
*E. kobei*	≥2	*E. coli*	≥2	*P. oryzihabitans*	≥2	*P. oryzihabitans*	≥2	*P. aeruginosa*	≥2	*K. cowanii*	≥2	*R. ornithinolytica*	≥2		
*E. kobei*	≥2	*P. oryzihabitans*	≥2	*P. oryzihabitans*	≥2	*P. oryzihabitans*	≥2	*P. aeruginosa*	≥2	*K. cowanii*	≥2	*M. luteus*	≥2		
*E. kobei*		*P. oryzihabitans*		*P. oryzihabitans*		*P. oryzihabitans*		*P. aeruginosa*		*P. agglomarans*		*M. luteus*			
*E. coli*		*P. oryzihabitans*		*P. oryzihabitans*		*P. oryzihabitans*		*P. aeruginosa*		*P. agglomarans*		*M. luteus*			

## Data Availability

The original contributions presented in the study are included in the article. Further inquiries can be directed to the corresponding author.

## Supplementary Materials

The following supporting information can be downloaded at: https://www.mdpi.com/article/10.3390/microorganisms13102267/s1, Files S1 and S2: Results of MaldI-Tof MS.
