# Supplementary material for: Short Communication: Evaluation of MALDI-TOF and Sequencing Technique as Typing Tools for Enterobacteriaceae Bacteria from Raw Milk of Dairy Cows with Subclinical Mastitis
Source: Microorganisms. 2025 Sep 27;13(10):2267. doi: 10.3390/microorganisms13102267 (PMC12566461; doi:10.3390/microorganisms13102267)
Supplement: Supplementary file 1 [file microorganisms-13-02267-s001.zip › microorganisms-3832023-supplementary/microorganisms-3832023-sup/Supplementary file 1.pdf]

Bruker MALDI Biotyper Identification Results

Run Info:

**Run Identifier:**	230526-1531-10014624052301
**Comment:**	146_240523_01 George Khasapane
**Operator:**	Admin@MBT-WIN10
**Run Creation Date/Time:**	2023-05-26T17:18:07.982
**Number of Tests:**	50
**Type:**	Standard
**BTS-QC:**	not present
**BTS-QC Position:**	
**Instrument ID:**	1865142.70324
**Server Version:**	4.1.100 (PYTH) 174 2019-06-158_01-16-09

Result Overview

Sample Name	Sample ID	Organism (best match)	Score Value	Organism (second-best match)	Score Value
A1 (+++) (A)	BTS (Standard)	Escherichia coli	2.27	Escherichia coli	2.23
A2 (+++) (A)	BTS (Standard)	Escherichia coli	2.29	Escherichia coli	2.24
A3 (+++) (A)	1 (Standard)	Enterococcus faecalis	2.36	Enterococcus faecalis	2.33
A4 (+++) (A)	1 (Standard)	Enterococcus faecalis	2.44	Enterococcus faecalis	2.39
A5 (+++) (A)	2 (Standard)	Pseudomonas oryzihabitans	2.27	Pseudomonas oryzihabitans	2.13
A6 (+++) (A)	2 (Standard)	Pseudomonas oryzihabitans	2.23	Pseudomonas oryzihabitans	2.13
A7 (+++) (A)	3 (Standard)	Enterococcus faecium	2.43	Enterococcus faecium	2.43

Result overview table--continued on next page

Result overview table--continued from previous page

Sample Name	Sample ID	Organism (best match)	Score Value	Organism (second-best match)	Score Value
A8 (+++) (A)	3 (Standard)	Enterococcus faecium	2.51	Enterococcus faecium	2.50
A9 (+++) (A)	4 (Standard)	Pseudomonas oryzihabitans	2.23	Pseudomonas oryzihabitans	2.18
A10 (+++) (A)	4 (Standard)	Pseudomonas oryzihabitans	2.24	Pseudomonas oryzihabitans	2.21
A11 (+++) (A)	5 (Standard)	Kosakonia cowanii	2.30	Kosakonia cowanii	2.23
A12 (+++) (A)	5 (Standard)	Kosakonia cowanii	2.32	Kosakonia cowanii	2.29
B1 (+++) (A)	6 (Standard)	Pseudomonas oryzihabitans	2.23	Pseudomonas oryzihabitans	2.14
B2 (+++) (A)	6 (Standard)	Pseudomonas oryzihabitans	2.22	Pseudomonas oryzihabitans	2.11
B3 (+++) (A)	7 (Standard)	Pseudomonas oryzihabitans	2.27	Pseudomonas oryzihabitans	2.13
B4 (+++) (A)	7 (Standard)	Pseudomonas oryzihabitans	2.31	Pseudomonas oryzihabitans	2.15
B5 (+++) (A)	8 (Standard)	Pseudomonas oryzihabitans	2.23	Pseudomonas oryzihabitans	2.09
B6 (+++) (A)	8 (Standard)	Pseudomonas oryzihabitans	2.25	Pseudomonas oryzihabitans	2.21
B7 (+++) (A)	9 (Standard)	Pseudomonas oryzihabitans	2.32	Pseudomonas oryzihabitans	2.11
B8 (+++) (A)	9 (Standard)	Pseudomonas oryzihabitans	2.21	Pseudomonas oryzihabitans	2.15
B9 (+++) (A)	10 (Standard)	Pantoea agglomerans	2.08	Pantoea agglomerans	1.98
B10 (+++) (A)	10 (Standard)	Pantoea agglomerans	2.01	Pantoea agglomerans	1.92
B11 (+++) (A)	11 (Standard)	Pseudomonas oryzihabitans	2.28	Pseudomonas oryzihabitans	2.15

Result overview table--continued on next page

Result overview table--continued from previous page

Sample Name	Sample ID	Organism (best match)	Score Value	Organism (second-best match)	Score Value
B12 (+++) (A)	11 (Standard)	Pseudomonas oryzihabitans	2.24	Pseudomonas oryzihabitans	2.21
C1 (+++) (A)	12 (Standard)	Pantoea agglomerans	2.05	Pantoea agglomerans	1.91
C2 (+++) (A)	12 (Standard)	Pantoea agglomerans	2.00	Pantoea agglomerans	1.89
C3 (+++) (A)	13 (Standard)	Pseudomonas oryzihabitans	2.35	Pseudomonas oryzihabitans	2.23
C4 (+++) (A)	13 (Standard)	Pseudomonas oryzihabitans	2.31	Pseudomonas oryzihabitans	2.16
C5 (+++) (A)	14 (Standard)	Pseudomonas oryzihabitans	2.25	Pseudomonas oryzihabitans	2.12
C6 (+++) (A)	14 (Standard)	Pseudomonas oryzihabitans	2.10	Pseudomonas oryzihabitans	1.97
C7 (+++) (A)	15 (Standard)	Pseudomonas oryzihabitans	2.20	Pseudomonas oryzihabitans	2.03
C8 (+++) (A)	15 (Standard)	Pseudomonas oryzihabitans	2.05	Pseudomonas oryzihabitans	2.04
C9 (+++) (A)	16 (Standard)	Pseudomonas oryzihabitans	2.12	Pseudomonas oryzihabitans	1.96
C10 (+++) (A)	16 (Standard)	Pseudomonas oryzihabitans	2.33	Pseudomonas oryzihabitans	2.07
C11 (+++) (A)	17 (Standard)	Enterobacter kobei typed as KPC positive	2.50	Enterobacter kobei	2.30
C12 (+++) (A)	17 (Standard)	Enterobacter kobei typed as KPC positive	2.45	Enterobacter kobei	2.28
D1 (+++) (A)	18 (Standard)	Pseudomonas aeruginosa	2.24	Pseudomonas aeruginosa	2.11
D2 (+++) (A)	18 (Standard)	Pseudomonas aeruginosa	2.34	Pseudomonas aeruginosa	2.13
D3 (+++) (A)	19 (Standard)	Pseudomonas oryzihabitans	2.23	Pseudomonas oryzihabitans	2.14

Result overview table--continued on next page

Result overview table--continued from previous page					
**Sample Name**	**Sample ID**	**Organism (best match)**	**Score Value**	**Organism (second-best match)**	**Score Value**
D4 (+++) (A)	19 (Standard)	Pseudomonas oryzihabitans	2.21	Pseudomonas oryzihabitans	2.16
D5 (+++) (A)	20 (Standard)	Pseudomonas oryzihabitans	2.21	Pseudomonas oryzihabitans	2.18
D6 (+++) (A)	20 (Standard)	Pseudomonas oryzihabitans	2.23	Pseudomonas oryzihabitans	2.07
D7 (+++) (A)	21 (Standard)	Pseudomonas oryzihabitans	2.22	Pseudomonas oryzihabitans	2.14
D8 (+++) (A)	21 (Standard)	Pseudomonas oryzihabitans	2.17	Pseudomonas oryzihabitans	2.06
D9 (+++) (B)	22 (Standard)	Raoultella ornithinolytica	2.45	Raoultella planticola	2.40
D10 (+++) (B)	22 (Standard)	Raoultella planticola	2.45	Raoultella ornithinolytica	2.44
D11 (+++) (A)	23 (Standard)	Enterobacter kobei	2.42	Enterobacter kobei	2.38
D12 (+++) (A)	23 (Standard)	Enterobacter kobei	2.39	Enterobacter kobei	2.34
E1 (+++) (A)	24 (Standard)	Pseudomonas oryzihabitans	2.30	Pseudomonas oryzihabitans	2.19
E2 (+++) (A)	24 (Standard)	Pseudomonas oryzihabitans	2.28	Pseudomonas oryzihabitans	2.13

Matching Hints

Matched Pattern	Comment
Citrobacter braakii 20663_2 CHB	is a member of Citrobacter freundii complex. Species braakii / freundii / gillenii / murliniae / rodentium / sedlakii / werkmannii / youngae of the genus Citrobacter have very similar patterns: Therefore distinguishing their species is difficult.
Citrobacter braakii 9314_2 CHB	is a member of Citrobacter freundii complex. Species braakii / freundii / gillenii / murliniae / rodentium / sedlakii / werkmannii / youngae of the genus Citrobacter have very similar patterns: Therefore distinguishing their species is difficult.
Cronobacter sp DSM 18706T DSM	Cronobacter can only be identified on genus level.
Cronobacter sp DSM 21870T DSM	Cronobacter can only be identified on genus level.
Cronobacter sp LMG 2758 LMG	Cronobacter can only be identified on genus level.
Cronobacter sp LMG 2789 LMG	Cronobacter can only be identified on genus level.
Enterobacter cloacae 13159_1 CHB	is a member of Enterobacter cloacae complex
Enterobacter cloacae 20105_2 CHB	is a member of Enterobacter cloacae complex
Enterobacter cloacae MB11506_1 CHB	is a member of Enterobacter cloacae complex
Enterobacter kobei C49 ADRIA	is a member of Enterobacter cloacae complex
Enterobacter kobei S58 ADRIA	is a member of Enterobacter cloacae complex
Escherichia coli ATCC 25922 CHB	closely related to Shigella / Escherichia fergusonii and not definitely distinguishable at the moment
Escherichia coli ATCC 25922 THL	closely related to Shigella / Escherichia fergusonii and not definitely distinguishable at the moment
Escherichia coli ATCC 35218 CHB	closely related to Shigella / Escherichia fergusonii and not definitely distinguishable at the moment
Escherichia coli DH5alpha BRL	closely related to Shigella / Escherichia fergusonii and not definitely distinguishable at the moment
Escherichia coli DSM 1103_QC DSM	closely related to Shigella / Escherichia fergusonii and not definitely distinguishable at the moment

Matching Hints table--continued on next page

Matched Pattern	Comment
Matching Hints table--continued from previous page	
Escherichia coli DSM 1576 DSM	closely related to Shigella / Escherichia fergusonii and not definitely distinguishable at the moment
Escherichia coli DSM 30083T HAM	closely related to Shigella / Escherichia fergusonii and not definitely distinguishable at the moment
Escherichia coli DSM 682 DSM	closely related to Shigella / Escherichia fergusonii and not definitely distinguishable at the moment
Escherichia coli ESBL_EA_RSS_1528T CHB	closely related to Shigella / Escherichia fergusonii and not definitely distinguishable at the moment
Escherichia coli MB11464_1 CHB	closely related to Shigella / Escherichia fergusonii and not definitely distinguishable at the moment
Escherichia coli Nissl VML	closely related to Shigella / Escherichia fergusonii and not definitely distinguishable at the moment
Escherichia coli RV412_A1_2010_06a LBK	closely related to Shigella / Escherichia fergusonii and not definitely distinguishable at the moment
Klebsiella oxytoca ATCC 700324 THL	Klebsiella oxytoca and species ornithinolytica / planticola / terrigena of the genus Raoultella have very similar patterns: Therefore distinguishing their species is difficult.
Klebsiella pneumoniae ssp pneumoniae 9295_1 CHB	closely related to Klebsiella variicola
Pantoea agglomerans CCM 2406 CCM	synonym of Erwinia herbicola
Pantoea agglomerans CCM 298 CCM	synonym of Erwinia herbicola
Pantoea agglomerans CCM 4412 CCM	synonym of Erwinia herbicola
Pantoea agglomerans CCM 4413 CCM	synonym of Erwinia herbicola
Pantoea agglomerans DSM 30074 DSM	synonym of Erwinia herbicola
Pantoea agglomerans DSM 30077 DSM	synonym of Erwinia herbicola
Pantoea agglomerans DSM 3493T HAM	synonym of Erwinia herbicola
Matching Hints table--continued on next page	

Matching Hints table--continued from previous page

Matched Pattern	Comment
Pantoea agglomerans DSM 8570 DSM	synonym of Erwinia herbicola
Pantoea agglomerans IMV 8606 PAH	synonym of Erwinia herbicola
Pseudescherichia vulneris 106220 IMHM	synonym of Escherichia vulneris
Pseudescherichia vulneris CCUG 21149 CCUG	synonym of Escherichia vulneris
Pseudescherichia vulneris CCUG 23001 CCUG	synonym of Escherichia vulneris
Pseudescherichia vulneris CCUG 26554 CCUG	synonym of Escherichia vulneris
Pseudescherichia vulneris DSM 4564T DSM	synonym of Escherichia vulneris
Pseudescherichia vulneris V308 MCRF	synonym of Escherichia vulneris
Pseudomonas luteola DSM 6975T HAM	is a member of Pseudomonas stutzeri group
Pseudomonas monteilii DSM 14164T HAM	is a member of Pseudomonas putida group
Pseudomonas oryzihabitans CCUG 31383 CCUG	is a member of Pseudomonas putida group
Pseudomonas oryzihabitans CCUG 46912 CCUG	is a member of Pseudomonas putida group
Pseudomonas oryzihabitans CCUG 51430 CCUG	is a member of Pseudomonas putida group
Pseudomonas oryzihabitans CCUG 60244 CCUG	is a member of Pseudomonas putida group
Pseudomonas oryzihabitans CCUG 9468 CCUG	is a member of Pseudomonas putida group
Pseudomonas oryzihabitans DSM 6835T DSM	is a member of Pseudomonas putida group

Matching Hints table--continued on next page

Matching Hints table--continued from previous page

Matched Pattern	Comment
Pseudomonas oryzihabitans DSM 6835T HAM	is a member of Pseudomonas putida group
Pseudomonas putida B400 UFL	is a member of Pseudomonas putida group
Pseudomonas putida DSM 291T HAM	is a member of Pseudomonas putida group
Pseudomonas rhodesiae DSM 14020T HAM	is a member of Pseudomonas fluorescens group
Raoultella ornithinolytica CCUG 52805 CCUG	Klebsiella oxytoca and species ornithinolytica / planticola / terrigena of the genus Raoultella have very similar patterns: Therefore distinguishing their species is difficult.
Raoultella ornithinolytica DSM 7464T DSM	Klebsiella oxytoca and species ornithinolytica / planticola / terrigena of the genus Raoultella have very similar patterns: Therefore distinguishing their species is difficult.
Raoultella ornithinolytica MB_18887 CHB	Klebsiella oxytoca and species ornithinolytica / planticola / terrigena of the genus Raoultella have very similar patterns: Therefore distinguishing their species is difficult.
Raoultella ornithinolytica MHNC_19_3 ERL	Klebsiella oxytoca and species ornithinolytica / planticola / terrigena of the genus Raoultella have very similar patterns: Therefore distinguishing their species is difficult.
Raoultella ornithinolytica MHNC_46_4 ERL	Klebsiella oxytoca and species ornithinolytica / planticola / terrigena of the genus Raoultella have very similar patterns: Therefore distinguishing their species is difficult.
Raoultella ornithinolytica MHNC_57_7 ERL	Klebsiella oxytoca and species ornithinolytica / planticola / terrigena of the genus Raoultella have very similar patterns: Therefore distinguishing their species is difficult.
Raoultella planticola DSM 2688 DSM	Klebsiella oxytoca and species ornithinolytica / planticola / terrigena of the genus Raoultella have very similar patterns: Therefore distinguishing their species is difficult.
Raoultella planticola DSM 3069T DSM	Klebsiella oxytoca and species ornithinolytica / planticola / terrigena of the genus Raoultella have very similar patterns: Therefore distinguishing their species is difficult.
Raoultella planticola DSM 4617 DSM	Klebsiella oxytoca and species ornithinolytica / planticola / terrigena of the genus Raoultella have very similar patterns: Therefore distinguishing their species is difficult.
Raoultella planticola MHNC_28_1 ERL	Klebsiella oxytoca and species ornithinolytica / planticola / terrigena of the genus Raoultella have very similar patterns: Therefore distinguishing their species is difficult.

Matching Hints table--continued on next page

Matching Hints table--continued from previous page	
**Matched Pattern**	**Comment**
Raoultella planticola MHNC_SB2787 ERL	Klebsiella oxytoca and species ornithinolytica / planticola / terrigena of the genus Raoultella have very similar patterns: Therefore distinguishing their species is difficult.
Raoultella planticola VA04253_09 ERL	Klebsiella oxytoca and species ornithinolytica / planticola / terrigena of the genus Raoultella have very similar patterns: Therefore distinguishing their species is difficult.
Raoultella planticola VA3440_3_09 ERL	Klebsiella oxytoca and species ornithinolytica / planticola / terrigena of the genus Raoultella have very similar patterns: Therefore distinguishing their species is difficult.
Salmonella sp (choleraesuis) 08 LAL	Salmonella can only be identified on genus level.

Meaning of Score Values

Range	Interpretation	Symbols	Color
2.00 - 3.00	High-confidence identification	(+++)	green
1.70 - 1.99	Low-confidence identification	(+)	yellow
0.00 - 1.69	No Organism Identification Possible	(-)	red

Meaning of Consistency Categories (A - C)

Category	Interpretation
(A)	**High consistency:** The best match is a high-confidence identification. The second-best match is (1) a high-confidence identification in which the species is identical to the best match, (2) a low-confidence identification in which the species or genus is identical to the best match, or (3) a non-identification.
(B)	**Low consistency:** The requirements for high consistency are not met. The best match is a high- or low-confidence identification. The second-best match is (1) a high- or low-confidence identification in which the genus is identical to the best match or (2) a non-identification.
(C)	**No consistency:** The requirements for high or low consistency are not met.
