# Supplementary material for: Short Communication: Evaluation of MALDI-TOF and Sequencing Technique as Typing Tools for Enterobacteriaceae Bacteria from Raw Milk of Dairy Cows with Subclinical Mastitis
Source: Microorganisms. 2025 Sep 27;13(10):2267. doi: 10.3390/microorganisms13102267 (PMC12566461; doi:10.3390/microorganisms13102267)
Supplement: Supplementary file 1 [file microorganisms-13-02267-s001.zip › microorganisms-3832023-supplementary/microorganisms-3832023-sup/Supplementary file 2.pdf]

Bruker MALDI Biotyper Identification Results

Run Info:

**Run Identifier:**	230530-1309-10014630052301
**Comment:**	146_300523_01 George Khasapane
**Operator:**	Admin@MBT-WIN10
**Run Creation Date/Time:**	2023-05-30T18:32:58.731
**Number of Tests:**	96
**Type:**	Standard
**BTS-QC:**	not present
**BTS-QC Position:**	
**Instrument ID:**	1865142.70324
**Server Version:**	4.1.100 (PYTH) 174 2019-06-158_01-16-09

Result Overview

Sample Name	Sample ID	Organism (best match)	Score Value	Organism (second-best match)	Score Value
A1 (+++) (A)	BTS (Standard)	Escherichia coli	2.22	Escherichia coli	2.14
A2 (+++) (A)	BTS (Standard)	Escherichia coli	2.28	Escherichia coli	2.22
A3 (+++) (A)	25 (Standard)	Escherichia coli	2.44	Escherichia coli	2.40
A4 (+++) (A)	25 (Standard)	Escherichia coli	2.42	Escherichia coli	2.34
A5 (+++) (A)	26 (Standard)	Enterobacter kobei typed as KPC positive	2.48	Enterobacter kobei	2.19
A6 (+++) (A)	26 (Standard)	Enterobacter kobei	2.39	Enterobacter kobei	2.33
A7 (+++) (A)	27 (Standard)	Pseudomonas aeruginosa	2.23	Pseudomonas aeruginosa	2.14

Result overview table--continued on next page

Result overview table--continued from previous page

Sample Name	Sample ID	Organism (best match)	Score Value	Organism (second-best match)	Score Value
A8 (+++) (A)	27 (Standard)	Pseudomonas aeruginosa	2.31	Pseudomonas aeruginosa	2.19
A9 (+++) (A)	28 (Standard)	Pseudomonas aeruginosa	2.10	Pseudomonas aeruginosa	2.09
A10 (+++) (A)	28 (Standard)	Pseudomonas aeruginosa	2.14	Pseudomonas aeruginosa	2.05
A11 (+++) (A)	29 (Standard)	Pseudomonas aeruginosa	2.10	Pseudomonas aeruginosa	2.02
A12 (+++) (A)	29 (Standard)	Pseudomonas aeruginosa	2.19	Pseudomonas aeruginosa	2.07
B1 (+++) (A)	30 (Standard)	Enterococcus faecalis	2.37	Enterococcus faecalis	2.32
B2 (+++) (A)	30 (Standard)	Enterococcus faecalis	2.42	Enterococcus faecalis	2.40
B3 (+++) (A)	31 (Standard)	Enterococcus faecium	2.48	Enterococcus faecium	2.42
B4 (+++) (A)	31 (Standard)	Enterococcus faecium	2.54	Enterococcus faecium	2.44
B5 (+) (B)	32 (Standard)	Pantoea agglomerans	1.89	Pantoea agglomerans	1.78
B6 (+) (B)	32 (Standard)	Pantoea agglomerans	1.94	Pantoea agglomerans	1.80
B7 (+) (B)	33 (Standard)	Pseudomonas oryzihabitans	1.76	Pseudomonas oryzihabitans	1.74
B8 (+) (B)	33 (Standard)	Pseudomonas oryzihabitans	1.80	**No Organism Identification Possible**	1.62
B9 (+++) (A)	34 (Standard)	Pseudomonas oryzihabitans	2.21	Pseudomonas oryzihabitans	2.12
B10 (+++) (A)	34 (Standard)	Pseudomonas oryzihabitans	2.26	Pseudomonas oryzihabitans	2.04
B11 (+++) (A)	35 (Standard)	Enterobacter kobei typed as KPC positive	2.49	Enterobacter kobei	2.24

Result overview table--continued on next page

Result overview table--continued from previous page

Sample Name	Sample ID	Organism (best match)	Score Value	Organism (second-best match)	Score Value
B12 (+++) (A)	35 (Standard)	Enterobacter kobei	2.44	Enterobacter kobei	2.34
C1 (+++) (A)	36 (Standard)	Pseudomonas oryzihabitans	2.25	Pseudomonas oryzihabitans	2.14
C2 (+++) (A)	36 (Standard)	Pseudomonas oryzihabitans	2.34	Pseudomonas oryzihabitans	2.07
C3 (+++) (A)	37 (Standard)	Kosakonia cowanii	2.08	Kosakonia cowanii	2.06
C4 (+++) (A)	37 (Standard)	Kosakonia cowanii	2.27	Kosakonia cowanii	2.22
C5 (+++) (A)	38 (Standard)	Pseudomonas oryzihabitans	2.30	Pseudomonas oryzihabitans	2.09
C6 (+++) (A)	38 (Standard)	Pseudomonas oryzihabitans	2.16	Pseudomonas oryzihabitans	2.00
C7 (+++) (A)	39 (Standard)	Pantoea agglomerans	2.01	Pantoea agglomerans	1.96
C8 (+++) (A)	39 (Standard)	Pantoea agglomerans	2.04	Pantoea agglomerans	1.82
C9 (+++) (A)	40 (Standard)	Pantoea agglomerans	2.09	Pantoea agglomerans	1.99
C10 (+++) (A)	40 (Standard)	Pantoea agglomerans	2.07	Pantoea agglomerans	1.87
C11 (+++) (A)	41 (Standard)	Enterococcus faecalis	2.41	Enterococcus faecalis	2.41
C12 (+++) (A)	41 (Standard)	Enterococcus faecalis	2.43	Enterococcus faecalis	2.39
D1 (+++) (A)	42 (Standard)	Micrococcus luteus	2.31	Micrococcus luteus	2.29
D2 (+++) (A)	42 (Standard)	Micrococcus luteus	2.32	Micrococcus luteus	2.25
D3 (+++) (A)	43 (Standard)	Raoultella ornithinolytica	2.35	Raoultella ornithinolytica	2.23

Result overview table--continued on next page

Result overview table--continued from previous page

Sample Name	Sample ID	Organism (best match)	Score Value	Organism (second-best match)	Score Value
D4 (+++) (A)	43 (Standard)	Raoultella ornithinolytica	2.29	Raoultella ornithinolytica	2.27
D5 (+++) (A)	44 (Standard)	Pseudomonas oryzihabitans	2.26	Pseudomonas oryzihabitans	2.18
D6 (+++) (A)	44 (Standard)	Pseudomonas oryzihabitans	2.14	Pseudomonas oryzihabitans	2.07
D7 (+++) (A)	45 (Standard)	Escherichia coli	2.43	Escherichia coli	2.31
D8 (+++) (A)	45 (Standard)	Escherichia coli	2.42	Escherichia coli	2.35
D9 (+++) (A)	46 (Standard)	Pseudomonas aeruginosa	2.18	Pseudomonas aeruginosa	2.07
D10 (+++) (A)	46 (Standard)	Pseudomonas aeruginosa	2.11	Pseudomonas aeruginosa	2.03
D11 (+++) (B)	47 (Standard)	Raoultella ornithinolytica	2.45	Raoultella ornithinolytica	2.44
D12 (+++) (B)	47 (Standard)	Raoultella ornithinolytica	2.37	Raoultella planticola	2.35
E1 (+++) (A)	48 (Standard)	Kosakonia cowanii	2.07	Kosakonia cowanii	2.02
E2 (+++) (A)	48 (Standard)	Kosakonia cowanii	2.16	Kosakonia cowanii	2.14
E3 (+) (B)	49 (Standard)	Mesobacillus thioparans	1.70	**No Organism Identification Possible**	1.68
E4 (+) (B)	49 (Standard)	Mesobacillus subterraneus	1.77	**No Organism Identification Possible**	1.62
E5 (+++) (A)	50 (Standard)	Pseudomonas oryzihabitans	2.28	Pseudomonas oryzihabitans	2.06
E6 (+++) (A)	50 (Standard)	Pseudomonas oryzihabitans	2.32	Pseudomonas oryzihabitans	2.16
E7 (+++) (A)	51 (Standard)	Pseudomonas aeruginosa	2.28	Pseudomonas aeruginosa	2.09

Result overview table--continued on next page

Result overview table--continued from previous page

Sample Name	Sample ID	Organism (best match)	Score Value	Organism (second-best match)	Score Value
E8 (+++) (A)	51 (Standard)	Pseudomonas aeruginosa	2.27	Pseudomonas aeruginosa	2.07
E9 (+++) (A)	52 (Standard)	Pseudomonas oryzihabitans	2.29	Pseudomonas oryzihabitans	2.22
E10 (+++) (A)	52 (Standard)	Pseudomonas oryzihabitans	2.18	Pseudomonas oryzihabitans	2.15
E11 (+++) (A)	53 (Standard)	Pseudomonas oryzihabitans	2.06	Pseudomonas oryzihabitans	2.04
E12 (+++) (A)	53 (Standard)	Pseudomonas oryzihabitans	2.20	Pseudomonas oryzihabitans	2.10
F1 (+++) (A)	54 (Standard)	Enterococcus faecium	2.54	Enterococcus faecium	2.51
F2 (+++) (A)	54 (Standard)	Enterococcus faecium	2.55	Enterococcus faecium	2.48
F3 (+++) (A)	55 (Standard)	Escherichia coli	2.34	Escherichia coli	2.31
F4 (+++) (A)	55 (Standard)	Escherichia coli	2.41	Escherichia coli	2.38
F5 (+++) (A)	56 (Standard)	Pseudomonas aeruginosa	2.22	Pseudomonas aeruginosa	2.10
F6 (+++) (A)	56 (Standard)	Pseudomonas aeruginosa	2.26	Pseudomonas aeruginosa	2.09
F7 (+++) (A)	57 (Standard)	Raoultella ornithinolytica	2.39	Raoultella ornithinolytica	2.37
F8 (+++) (A)	57 (Standard)	Raoultella ornithinolytica	2.27	Raoultella ornithinolytica	2.23
F9 (+++) (A)	58 (Standard)	Escherichia coli	2.45	Escherichia coli	2.42
F10 (+++) (A)	58 (Standard)	Escherichia coli	2.47	Escherichia coli	2.42
F11 (+++) (A)	59 (Standard)	Micrococcus luteus	2.28	Micrococcus luteus	2.18

Result overview table--continued on next page

Result overview table--continued from previous page

Sample Name	Sample ID	Organism (best match)	Score Value	Organism (second-best match)	Score Value
F12 (+++) (A)	59 (Standard)	Micrococcus luteus	2.29	Micrococcus luteus	2.21
G1 (+++) (A)	60 (Standard)	Escherichia coli	2.43	Escherichia coli	2.37
G2 (+++) (A)	60 (Standard)	Escherichia coli	2.40	Escherichia coli	2.39
G3 (+++) (A)	61 (Standard)	Micrococcus luteus	2.24	Micrococcus luteus	2.18
G4 (+++) (A)	61 (Standard)	Micrococcus luteus	2.27	Micrococcus luteus	2.26
G5 (+++) (A)	62 (Standard)	Pseudomonas aeruginosa	2.35	Pseudomonas aeruginosa	2.21
G6 (+++) (A)	62 (Standard)	Pseudomonas aeruginosa	2.31	Pseudomonas aeruginosa	2.25
G7 (+++) (A)	63 (Standard)	Pseudomonas oryzihabitans	2.27	Pseudomonas oryzihabitans	2.20
G8 (+++) (A)	63 (Standard)	Pseudomonas oryzihabitans	2.32	Pseudomonas oryzihabitans	2.23
G9 (+++) (A)	64 (Standard)	Kosakonia cowanii	2.39	Kosakonia cowanii	2.26
G10 (+++) (A)	64 (Standard)	Kosakonia cowanii	2.44	Kosakonia cowanii	2.40
G11 (+++) (A)	65 (Standard)	Pseudomonas oryzihabitans	2.28	Pseudomonas oryzihabitans	2.03
G12 (+++) (A)	65 (Standard)	Pseudomonas oryzihabitans	2.26	Pseudomonas oryzihabitans	2.01
H1 (+++) (A)	66 (Standard)	Escherichia coli	2.51	Escherichia coli	2.47
H2 (+++) (A)	66 (Standard)	Escherichia coli	2.32	Escherichia coli	2.27
H3 (+++) (A)	67 (Standard)	Escherichia coli	2.46	Escherichia coli	2.42

Result overview table--continued on next page

Result overview table--continued from previous page					
**Sample Name**	**Sample ID**	**Organism (best match)**	**Score Value**	**Organism (second-best match)**	**Score Value**
H4 (+++) (A)	67 (Standard)	Escherichia coli	2.54	Escherichia coli	2.48
H5 (+++) (A)	68 (Standard)	Escherichia coli	2.31	Escherichia coli	2.28
H6 (+++) (A)	68 (Standard)	Escherichia coli	2.39	Escherichia coli	2.36
H7 (+++) (A)	69 (Standard)	Enterobacter kobei typed as KPC positive	2.31	Enterobacter kobei	2.02
H8 (+++) (A)	69 (Standard)	Enterobacter kobei	2.23	Enterobacter kobei	2.03
H9 (+++) (A)	70 (Standard)	Enterobacter kobei	2.17	Enterobacter kobei	2.07
H10 (+++) (A)	70 (Standard)	Enterobacter kobei	2.20	Enterobacter kobei	2.08
H11 (+++) (A)	71 (Standard)	Pseudomonas aeruginosa	2.25	Pseudomonas aeruginosa	2.11
H12 (+++) (A)	71 (Standard)	Pseudomonas aeruginosa	2.25	Pseudomonas aeruginosa	2.13

Matching Hints

Matched Pattern	Comment
Aeribacillus pallidus DSM 28917 DSM	For the species composti / pallidus of the genus Aeribacillus the 16S rRNA gene sequences are very similar. Therefore distinguishing the mentioned species is difficult.
Bacillus marisflavi DSM 16204T DSM	The quality of spectra (score) depends on the degree of sporulation: Use fresh material.
Bacillus thermoamylovorans 10w413986 RLH	The quality of spectra (score) depends on the degree of sporulation: Use fresh material.
Citrobacter braakii 20663_2 CHB	is a member of Citrobacter freundii complex. Species braakii / freundii / gillenii / murliniae / rodentium / sedlakii / werkmannii / youngae of the genus Citrobacter have very similar patterns: Therefore distinguishing their species is difficult.
Citrobacter freundii 13158_2 CHB	is a member of Citrobacter freundii complex. Species braakii / freundii / gillenii / murliniae / rodentium / sedlakii / werkmannii / youngae of the genus Citrobacter have very similar patterns: Therefore distinguishing their species is difficult.
Cronobacter sp CCM 3461 CCM	Cronobacter can only be identified on genus level.
Cronobacter sp CCM 3479 CCM	Cronobacter can only be identified on genus level.
Cronobacter sp DSM 18705T DSM	Cronobacter can only be identified on genus level.
Cronobacter sp DSM 18706T DSM	Cronobacter can only be identified on genus level.
Cronobacter sp DSM 21870T DSM	Cronobacter can only be identified on genus level.
Cronobacter sp LMG 2758 LMG	Cronobacter can only be identified on genus level.
Cronobacter sp LMG 2786 LMG	Cronobacter can only be identified on genus level.
Cronobacter sp LMG 2789 LMG	Cronobacter can only be identified on genus level.
Cronobacter sp LMG 2790 LMG	Cronobacter can only be identified on genus level.
Enterobacter cloacae 13159_1 CHB	is a member of Enterobacter cloacae complex

Matching Hints table--continued on next page

Matching Hints table--continued from previous page	
**Matched Pattern**	**Comment**
Enterobacter cloacae 20105_2 CHB	is a member of Enterobacter cloacae complex
Enterobacter cloacae MB11506_1 CHB	is a member of Enterobacter cloacae complex
Enterobacter cloacae ssp dissolvens DSM 16657T DSM	is a member of Enterobacter cloacae complex
Enterobacter kobei C49 ADRIA	is a member of Enterobacter cloacae complex
Enterobacter kobei S58 ADRIA	is a member of Enterobacter cloacae complex
Escherichia coli ATCC 25922 CHB	closely related to Shigella / Escherichia fergusonii and not definitely distinguishable at the moment
Escherichia coli ATCC 25922 THL	closely related to Shigella / Escherichia fergusonii and not definitely distinguishable at the moment
Escherichia coli ATCC 35218 CHB	closely related to Shigella / Escherichia fergusonii and not definitely distinguishable at the moment
Escherichia coli DH5alpha BRL	closely related to Shigella / Escherichia fergusonii and not definitely distinguishable at the moment
Escherichia coli DSM 1103_QC DSM	closely related to Shigella / Escherichia fergusonii and not definitely distinguishable at the moment
Escherichia coli DSM 1576 DSM	closely related to Shigella / Escherichia fergusonii and not definitely distinguishable at the moment
Escherichia coli DSM 30083T HAM	closely related to Shigella / Escherichia fergusonii and not definitely distinguishable at the moment
Escherichia coli DSM 682 DSM	closely related to Shigella / Escherichia fergusonii and not definitely distinguishable at the moment
Escherichia coli ESBL_EA_RSS_1528T CHB	closely related to Shigella / Escherichia fergusonii and not definitely distinguishable at the moment
Escherichia coli MB11464_1 CHB	closely related to Shigella / Escherichia fergusonii and not definitely distinguishable at the moment
Escherichia coli Nissl VML	closely related to Shigella / Escherichia fergusonii and not definitely distinguishable at the moment
Escherichia coli RV412_A1_2010_06a LBK	closely related to Shigella / Escherichia fergusonii and not definitely distinguishable at the moment
Matching Hints table--continued on next page	

Matching Hints table--continued from previous page

Matched Pattern	Comment
Escherichia coli W3350 MMG	closely related to Shigella / Escherichia fergusonii and not definitely distinguishable at the moment
Escherichia fergusonii DSM 13698T HAM	closely related to Shigella / Escherichia coli and not definitely distinguishable at the moment
Fictibacillus arsenicus DSM 15822T DSM	The quality of spectra (score) depends on the degree of sporulation: Use fresh material.
Klebsiella aerogenes DSM 12058 DSM	synonym of Enterobacter aerogenes
Klebsiella oxytoca ATCC 700324 THL	Klebsiella oxytoca and species ornithinolytica / planticola / terrigena of the genus Raoultella have very similar patterns: Therefore distinguishing their species is difficult.
Klebsiella pneumoniae ssp pneumoniae 9295_1 CHB	closely related to Klebsiella variicola
Lactobacillus amylovorus DSM 16698 DSM	Species amylovorus / kitasatonis of the genus Lactobacillus have very similar patterns: Therefore distinguishing their species is difficult.
Mesobacillus jeotgali DSM 18226T DSM	Synonym of Bacillus jeotgali. The quality of spectra (score) depends on the degree of sporulation: Use fresh material.
Mesobacillus subterraneus 58786 RQCL	Synonym of Bacillus subterraneus. The quality of spectra (score) depends on the degree of sporulation: Use fresh material.
Mesobacillus thioparans CIP 109765T CIP	Synonym of Bacillus thioparans. The quality of spectra (score) depends on the degree of sporulation: Use fresh material.
Mesobacillus thioparans CIP 109765T CIP_2	Synonym of Bacillus thioparans. The quality of spectra (score) depends on the degree of sporulation: Use fresh material.
Pantoea agglomerans CCM 2406 CCM	synonym of Erwinia herbicola
Pantoea agglomerans CCM 298 CCM	synonym of Erwinia herbicola
Pantoea agglomerans CCM 4412 CCM	synonym of Erwinia herbicola
Pantoea agglomerans CCM 4413 CCM	synonym of Erwinia herbicola
Pantoea agglomerans DSM 30074 DSM	synonym of Erwinia herbicola

Matching Hints table--continued on next page

Matched Pattern	Comment
Matching Hints table--continued from previous page	
Pantoea agglomerans DSM 30077 DSM	synonym of Erwinia herbicola
Pantoea agglomerans DSM 3493T HAM	synonym of Erwinia herbicola
Pantoea agglomerans DSM 8570 DSM	synonym of Erwinia herbicola
Pantoea agglomerans IMV 8456_2406 PAH	synonym of Erwinia herbicola
Pantoea agglomerans IMV 8606 PAH	synonym of Erwinia herbicola
Pantoea anthophila DSM 23080T DSM	For the species anthophila / deleyi / eucalypti of the genus Pantoea the 16S rRNA gene sequences are very similar. Therefore distinguishing the mentioned species is difficult.
Peribacillus muralis DSM 16288T DSM	Synonym of Bacillus muralis. The quality of spectra (score) depends on the degree of sporulation: Use fresh material.
Pseudescherichia vulneris 106220 IMHM	synonym of Escherichia vulneris
Pseudescherichia vulneris CCUG 21149 CCUG	synonym of Escherichia vulneris
Pseudescherichia vulneris CCUG 23001 CCUG	synonym of Escherichia vulneris
Pseudescherichia vulneris CCUG 26554 CCUG	synonym of Escherichia vulneris
Pseudescherichia vulneris DSM 4564T DSM	synonym of Escherichia vulneris
Pseudescherichia vulneris V308 MCRF	synonym of Escherichia vulneris
Pseudomonas corrugata DSM 7228T HAM	is a member of Pseudomonas fluorescens group
Pseudomonas luteola DSM 6975T HAM	is a member of Pseudomonas stutzeri group
Pseudomonas oryzihabitans CCUG 31383 CCUG	is a member of Pseudomonas putida group
Matching Hints table--continued on next page	

Matching Hints table--continued from previous page

Matched Pattern	Comment
Pseudomonas oryzihabitans CCUG 46912 CCUG	is a member of Pseudomonas putida group
Pseudomonas oryzihabitans CCUG 51430 CCUG	is a member of Pseudomonas putida group
Pseudomonas oryzihabitans CCUG 60244 CCUG	is a member of Pseudomonas putida group
Pseudomonas oryzihabitans CCUG 9468 CCUG	is a member of Pseudomonas putida group
Pseudomonas oryzihabitans DSM 6835T DSM	is a member of Pseudomonas putida group
Pseudomonas oryzihabitans DSM 6835T HAM	is a member of Pseudomonas putida group
Pseudomonas putida DSM 291T HAM	is a member of Pseudomonas putida group
Pseudomonas rhodesiae DSM 14020T HAM	is a member of Pseudomonas fluorescens group
Raoultella ornithinolytica CCUG 52805 CCUG	Klebsiella oxytoca and species ornithinolytica / planticola / terrigena of the genus Raoultella have very similar patterns: Therefore distinguishing their species is difficult.
Raoultella ornithinolytica CIP 103576 CIP	Klebsiella oxytoca and species ornithinolytica / planticola / terrigena of the genus Raoultella have very similar patterns: Therefore distinguishing their species is difficult.
Raoultella ornithinolytica DSM 7464T DSM	Klebsiella oxytoca and species ornithinolytica / planticola / terrigena of the genus Raoultella have very similar patterns: Therefore distinguishing their species is difficult.
Raoultella ornithinolytica DSM 7464T HAM	Klebsiella oxytoca and species ornithinolytica / planticola / terrigena of the genus Raoultella have very similar patterns: Therefore distinguishing their species is difficult.
Raoultella ornithinolytica MB_18887 CHB	Klebsiella oxytoca and species ornithinolytica / planticola / terrigena of the genus Raoultella have very similar patterns: Therefore distinguishing their species is difficult.
Raoultella ornithinolytica MHNC_19_3 ERL	Klebsiella oxytoca and species ornithinolytica / planticola / terrigena of the genus Raoultella have very similar patterns: Therefore distinguishing their species is difficult.

Matching Hints table--continued on next page

Matching Hints table--continued from previous page	
**Matched Pattern**	**Comment**
Raoultella ornithinolytica MHNC_46_4 ERL	Klebsiella oxytoca and species ornithinolytica / planticola / terrigena of the genus Raoultella have very similar patterns: Therefore distinguishing their species is difficult.
Raoultella ornithinolytica MHNC_57_7 ERL	Klebsiella oxytoca and species ornithinolytica / planticola / terrigena of the genus Raoultella have very similar patterns: Therefore distinguishing their species is difficult.
Raoultella ornithinolytica UR01120_09 ERL	Klebsiella oxytoca and species ornithinolytica / planticola / terrigena of the genus Raoultella have very similar patterns: Therefore distinguishing their species is difficult.
Raoultella planticola DSM 3069T DSM	Klebsiella oxytoca and species ornithinolytica / planticola / terrigena of the genus Raoultella have very similar patterns: Therefore distinguishing their species is difficult.
Raoultella planticola DSM 4617 DSM	Klebsiella oxytoca and species ornithinolytica / planticola / terrigena of the genus Raoultella have very similar patterns: Therefore distinguishing their species is difficult.
Raoultella planticola MHNC_28_1 ERL	Klebsiella oxytoca and species ornithinolytica / planticola / terrigena of the genus Raoultella have very similar patterns: Therefore distinguishing their species is difficult.
Raoultella planticola MHNC_SB2787 ERL	Klebsiella oxytoca and species ornithinolytica / planticola / terrigena of the genus Raoultella have very similar patterns: Therefore distinguishing their species is difficult.
Raoultella planticola VA04253_09 ERL	Klebsiella oxytoca and species ornithinolytica / planticola / terrigena of the genus Raoultella have very similar patterns: Therefore distinguishing their species is difficult.
Raoultella planticola VA3440_3_09 ERL	Klebsiella oxytoca and species ornithinolytica / planticola / terrigena of the genus Raoultella have very similar patterns: Therefore distinguishing their species is difficult.
Salmonella sp (choleraesuis) 08 LAL	Salmonella can only be identified on genus level.
Streptomyces collinus DSM 40129T DSM	Species of this genus have very similar patterns: Therefore distinguishing their species is difficult.

Meaning of Score Values

Range	Interpretation	Symbols	Color
2.00 - 3.00	High-confidence identification	(+++)	green
1.70 - 1.99	Low-confidence identification	(+)	yellow
0.00 - 1.69	No Organism Identification Possible	(-)	red

Meaning of Consistency Categories (A - C)

Category	Interpretation
(A)	**High consistency:** The best match is a high-confidence identification. The second-best match is (1) a high-confidence identification in which the species is identical to the best match, (2) a low-confidence identification in which the species or genus is identical to the best match, or (3) a non-identification.
(B)	**Low consistency:** The requirements for high consistency are not met. The best match is a high- or low-confidence identification. The second-best match is (1) a high- or low-confidence identification in which the genus is identical to the best match or (2) a non-identification.
(C)	**No consistency:** The requirements for high or low consistency are not met.
